# Epigenome-Wide DNA Methylation Profiling in Colorectal Cancer and Normal Adjacent Colon Using Infinium Human Methylation 450K

**DOI:** 10.3390/diagnostics12010198

**Published:** 2022-01-14

**Authors:** Rashidah Baharudin, Muhiddin Ishak, Azliana Muhamad Yusof, Sazuita Saidin, Saiful Effendi Syafruddin, Wan Fahmi Wan Mohamad Nazarie, Learn-Han Lee, Nurul-Syakima Ab Mutalib

**Affiliations:** 1 UKM Medical Molecular Biology Institute (UMBI), Universiti Kebangsaan Malaysia, Kuala Lumpur 56000, Malaysia; iedabaharudin90@gmail.com (R.B.); muhiddin@ppukm.ukm.edu.my (M.I.); sazuita@ukm.edu.my (S.S.); effendisy@ppukm.ukm.edu.my (S.E.S.); 2 Cytogenetics & Molecular Diagnostics Laboratory, Pantai Premier Pathology Sdn Bhd, Pandan Indah, Kuala Lumpur 55100, Malaysia; azlianayusof@gmail.com; 3 Faculty of Science and Natural Resources, Universiti Malaysia Sabah, Kota Kinabalu 88400, Malaysia; wanfahmi5785@gmail.com; 4 Novel Bacteria and Drug Discovery Research Group, Microbiome and Bioresource Research Strength, Jeffrey Cheah School of Medicine and Health Sciences, Monash University of Malaysia, Subang Jaya 47500, Malaysia; 5 Faculty of Health Sciences, Universiti Kebangsaan Malaysia, Kuala Lumpur 50300, Malaysia

**Keywords:** colorectal cancer, DNA methylation, adjacent normal colon, Infinium Human Methylation 450K, microarray

## Abstract

The aims were to profile the DNA methylation in colorectal cancer (CRC) and to explore cancer-specific methylation biomarkers. Fifty-four pairs of CRCs and the adjacent normal tissues were subjected to Infinium Human Methylation 450K assay and analysed using ChAMP R package. A total of 26,093 differentially methylated probes were identified, which represent 6156 genes; 650 probes were hypermethylated, and 25,443 were hypomethylated. Hypermethylated sites were common in CpG islands, while hypomethylated sites were in open sea. Most of the hypermethylated genes were associated with pathways in cancer, while the hypomethylated genes were involved in the PI3K-AKT signalling pathway. Among the identified differentially methylated probes, we found evidence of four potential probes in CRCs versus adjacent normal; *HOXA2* cg06786372, *OPLAH* cg17301223, cg15638338, and *TRIM31* cg02583465 that could serve as a new biomarker in CRC since these probes were aberrantly methylated in CRC as well as involved in the progression of CRC. Furthermore, we revealed the potential of promoter methylation *ADHFE1* cg18065361 in differentiating the CRC from normal colonic tissue from the integrated analysis. In conclusion, aberrant DNA methylation is significantly involved in CRC pathogenesis and is associated with gene silencing. This study reports several potential important methylated genes in CRC and, therefore, merit further validation as novel candidate biomarker genes in CRC.

## 1. Introduction

Colorectal cancer (CRC) is a leading cause of morbidity and cancer death worldwide. In Malaysia, CRC is identified as the most common cancer in men and the second most common cancer in women [1]. This disease is highly heterogeneous, with varying responses to cancer therapy and prognosis. The heterogeneity of CRC evolved from multiple pathways, including Chromosomal Instability (CIN), Microsatellite Instability (MSI), and CpG Island Methylator Phenotype (CIMP) [2]. Unlike other pathways, CIMP is an epigenetic mechanism that is more dynamic [3] and often reversible in the presence of inducing factors such as demethylating agents. The concept of CIMP was first introduced by Toyota et al. in 1999 and defined as widespread methylation in the CpG island of the genes [4]. The methylation process was described by the addition of the methyl group (CH_3_^−^) at the carbon 5 of the cytosine ring to form 5-methylcytosine, and the process is catalysed by the enzyme DNA methyltransferase (DNMT) in which S-adenosyl-methionine (SAM) acts as a methyl donor [5,6]. Aberrant methylation in CRC has correlated with the inactivation of tumour suppressor genes [7] and the activation of oncogenes [8] that function to control a variety of cellular processes, including apoptosis, proliferation, invasion, and migration [9]. Aberrant DNA methylation is a hallmark of cancer that occurs early in cancer development [10], increases with the progression of the disease, and is involved in the treatment response [11,12]. Therefore, DNA methylation may serve as a potential biomarker for cancer diagnosis, predicting patient prognosis, and monitoring response towards therapy. Several studies have been conducted to identify a methylation biomarker with high specificity and sensitivity to be used in the diagnosis of CRC. For instance, Freitas and his colleagues discovered promoter methylation of the three-genes panel (*MGMT*, *RASSF1A,* and *SEPT9*) in accurately diagnosed CRC with 96.6% sensitivity and 74% specificity of detection [13]. Furthermore, a meta-analysis from 38 studies has offered the potential diagnostic markers of hypermethylation *SFRP1*, *SFRP2*, *NDRG2,* and *VIM* genes in CRC patients [14].

As previously stated, the methylation status of specific genes may also predict the prognosis of the patients. An interesting study by Maija et al. 2013, discovered that the activation of oncogene *KRAS* along with promoter methylation of *CDKN2A* leads to more aggressive rectal cancers [15]. In addition, *CHFR* is another promising prognostic marker whereby promoter methylation of this gene indicates poor prognosis in stage II microsatellite stable CRC [16]. Numerous investigations have identified a therapeutic response mediated by DNA methylation [17,18,19]. In our previous research, we identified the aberrant methylation of five potential therapeutic targets, *CCNE1*, *CCNDBP1*, *PON3*, *CHL1*, and *DDX43*, involved in CRC chemoresistance [20]. Moreover, CRC patients with the hypermethylation of *NKX6.1* [21], *TFAP2E-DKK4* [22], and *IGFBP3* genes [23] were unresponsive to 5-FU chemotherapy treatment. Patients with unmethylated *RASSF1A* [24] and *SRBC* [18] were highly responsive to oxaliplatin chemotherapy drugs than patients who exhibited aberrant methylation of these genes. However, the methylation of *GPX3* was associated with oxaliplatin sensitivity [25]. The hypermethylation of *BNIP3* reduces the sensitivity of CRC towards irinotecan chemotherapeutic drugs [26].

DNA methylation has been recognised as a potential biomarker in CRC; however, only a few methylation markers are currently used in cancer diagnostics. DNA methylation-based biomarkers are still relatively new, hence, careful assessments of the potential biomarkers are required to further validate them prior to being used in clinical diagnostics. Therefore, in this study, we aim to profile the DNA methylation in CRC and explore cancer-specific methylation biomarkers to provide evidence that can support their use in clinical practice.

## 2. Materials and Methods

### 2.1. Clinical Specimens

Fifty-four pairs (*n* = 108) of CRC and the respective adjacent normal tissues were collected from the UKM Medical Center, Malaysia. This study was conducted following the recommendations and approval of the Universiti Kebangsaan Malaysia Research Ethics Committee (Reference number: UKM 1.5.3.5/244/UMBI-004-2012). All subjects gave written informed consent for their participation following the Declaration of Helsinki. The tissues were dissected, snap-frozen and stored in liquid nitrogen prior to sectioning. All sectioned tissues were stained with Hematoxylin and Eosin (H&E). Only cancer tissues that contained more than 80% cancerous cells and normal adjacent tissues with less than 20% necrosis were subjected to the next step. Genomic DNA from frozen tissues was extracted using the QIAamp DNA mini kit according to the manufacturer’s instructions. The quantification and purity of DNA for each sample were assessed using Qubit 2.0 fluorometer and Nanodrop 2000c Spectrometer (Thermo Fisher Scientific, Inc., Wilmington, DE, USA), respectively. Only samples with purity from 1.8 to 2.0 were selected for the microarray study.

### 2.2. DNA Methylation Profiling

Methylation profiling was performed on 108 samples (54 paired tumour–adjacent normal colon) using the Infinium Human Methylation 450K BeadChip, which covers 485,577 CpG dinucleotide sites distributed over the whole genome according to the manufacturer’s specification (Illumina, Inc., San Diego, CA, USA). Genomic DNA underwent bisulfite treatment to convert all unmethylated cytosine to uracil using EZ DNA methylation—Gold kit (Zymo Research, Inc., Irvine, CA, USA) following the manufacturer’s protocol prior to being subjected to profiling. Scanning of the BeadChips was performed on the iScan scanner (Illumina, Inc., San Diego, CA, USA).

### 2.3. Human Methylation 450K Data Analysis

The raw IDAT files were exported from the scanner, and quality control was performed using Genome Studio software version 2.0.4 (Illumina Inc.). The passed IDAT files from 108 samples were further analysed using the ChAMP R package [27] in a single analysis, and filters were applied to all datasets where CpG sites that had a detection *p*-value > 0.01 in each probe were excluded from further analysis. The data were normalised using the Peak-Based Correction (PBC) method [28] prior to the batch effect correction using ComBat [29]. The β-values were extracted, and statistical analysis was performed. The limma Bioconductor package was used to determine the differentially methylated CpG sites [30,31], and we applied Benjamini–Hochberg (BH) *p*-value < 0.05 to identify significant differentially methylated probes. Then, to determine the methylation status of the probes, we conducted further filtering based on the ∆β value of tumour versus normal, where ≥0.2 was considered hypermethylated and ∆β ≤ −0.2 was hypomethylated. The heatmap was generated using the online Morpheus tool from the Broad Institute [32].

### 2.4. Functional Enrichment Analyses of Differentially Methylated Genes

The DMGs were then subjected to gene ontology enrichment analysis using the bioinformatics analysis tool, DAVID Bioinformatics Resources 6.8 [33,34], to identify the pathways involved in the hypermethylated and hypomethylated genes. Adjusted *p*-values < 0.05 were used as the cut-off criterion.

### 2.5. Gene Expression Profile Analysis

The gene profiling analysis was performed using the level 3 IlluminaHiSeq RNAseqV2 mRNA dataset in patients diagnosed with CRC from The Cancer Genome Atlas (TCGA) data portal. A total of 157 samples were available for gene expression data, with 13 matched normal and tumour samples. The normalised RNAseq by Expectation-Maximization (RSEM) data was input into R programming software, and the limma Bioconductor package was subsequently used for the calculation of differentially expressed genes. The Benjamini–Hochberg (BH) [35] procedure was applied to identify significantly differentially expressed genes between CRC and normal colon tissues with the cut-off criterion of adjusted *p*-values < 0.05 and log2 fold change (FC) ≥ |1|.

### 2.6. Integrated Promoter Methylation and Gene Expression Profiling

In order to identify a set of genes whose expression is primarily and causally regulated by promoter DNA methylation in CRC, we performed an integrated analysis of genome-wide DNA methylation and a gene expression profile. The promoter region was defined as the genomic interval that begins 1500 bp upstream and 200 bp downstream of the transcription start site in the CG-rich region. Additionally, the gene expression data were classified into two groups according to their expression level in which differentially expressed genes were exhibited, log2 FC > 1 were considered upregulated, and log2 FC < −1 in CRC, compared to the normal colon, were considered downregulated. The promoter methylation mediated silencing genes were determined by overlapping the methylated promoter genes with the downregulated genes.

### 2.7. Protein-Protein Interaction

The interaction between the proteins encoded by top promoter methylated-silencing genes were determined using the Search Tool for the Retrieval of Interacting Genes (STRING) v11 database [36]. All parameters were set to defaults. In the search for the candidate biomarker, a few criteria were utilised, such as genes that posed a high level of methylation and low expression, genes that have strong interaction that can regulate other genes, as well as genes associated with predisposition to CRC.

### 2.8. Receiver Operating Characteristics (ROC) Curve of Genes

The diagnostic performance of the candidate biomarker was evaluated by the ROC curves. The area under the ROC curve (AUC) was constructed with a 95% confidence interval (95% CI) as an accuracy criterion for the examination of the candidate biomarker. The methylation value of candidate biomarkers in CRC cases was plotted against their corresponding control, and a perfect diagnostic marker had an AUC value of 1. All the analyses were generated using GraphPad Prism 8.0.2.

## 3. Results

### 3.1. Demography

Demography data of the 54 patients are presented in Table 1. The majority of the patients were female and above the age of 50 years old. Most of the patients were diagnosed with Duke’s B and positioned on the left side of the colon. Moreover, the majority of the tumour tissues were moderately differentiated.

### 3.2. Locations of Differentially Methylated Probes

**Figure 1:**
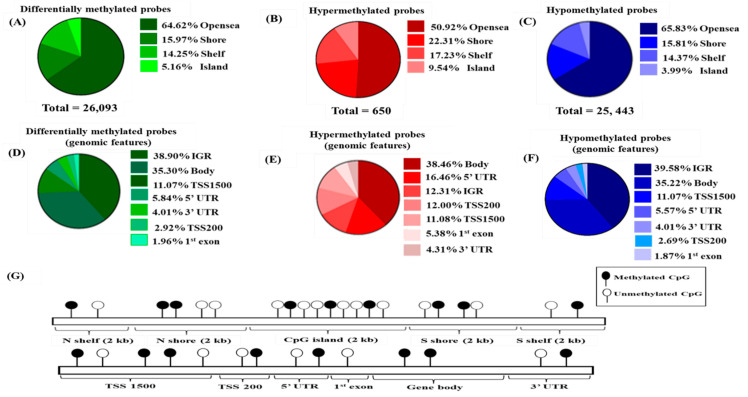
Differentially methylated probes in CRCs relative to its adjacent normal. (**A**) Distribution of significantly differentially methylated probes in a genomic region. (**B**) Hypermethylated probes and (**C**) Hypomethylated probes in genomic region. (**D**) Distribution of differentially methylated probes, (**E**) hypermethylated probes, (**F**) Hypomethylated probes with respect to genomic features, (**G**) distribution of methylation CpG sites in the human genome. The CpG island is surrounded by shores (within 2 kb sequence neighbouring the islands) with shelves flanked further from the shores. The open sea area is outside of the shelf region.

We compared the differential methylation status of 54 CRC tissue samples with the 54 adjacent cancer-free colonic tissue samples. In order to explore epigenome-wide methylation profiles, probe filtering was performed to identify the differentially methylated probes with a detection adjusted *p*-value < 0.01 after FDR correction. This resulted in 157,846 probes for the downstream analysis. These probes were further classified as hypermethylated or hypomethylated based on the absolute average β value difference (Δβ) at ≥0.2 between CRC and normal adjacent tissues. This value represents 20% change in the methylation level. A total of 26,093 probes were identified (Figure 1A). Of these, 650 probes were hypermethylated and 25,443 probes were hypomethylated. Then, we stratified the probes into CpG island, shores, shelves, and open sea regions. From the 650 differentially hypermethylated probes, 331 probes (50.92%) were located in the island region, accounting for half of the total number of probes, 145 probes (22.31%) were located on the shore, 112 probes (17.23%) were in the open sea, and the remaining 62 probes (9.54%) were located in the shelf region (Figure 1B). In contrast, most of the hypomethylated probes were in the open sea area of the genome (*n* = 16,749; 65.83%), followed by the shore, shelf, and island region with 4022 (15.81%), 3657 (14.37%), and 1015 (3.99%) probes, respectively (Figure 1C).

Meanwhile, categorization based on genomic features revealed that most differentially methylated probes did not belong to any gene. The majority of these probes were in the intergenic region (IGR) (*n* = 10,150; 38.90%), closely followed by the gene body (*n* = 9211; 35.30%), TSS1500 (*n* = 2888; 11.07%), 5′UTR (*n* = 1524; 5.84%), 3′UTR (*n* = 1047; 4.01%), TSS200 (*n* = 762; 2.92%), and 1st exon (*n* = 511; 1.96%), as illustrated in Figure 1D. On closer inspection, the majority of the significantly hypermethylated loci were in the body (*n* = 250; 38.46%), followed by 5′UTR (*n* = 107; 16.46%), the intergenic region (*n* = 80; 12.31%), closely followed by TSS200 (*n* = 78; 12%), TSS1500 (*n* = 72; 11.08%), 1st exon (*n* = 35; 5.38%), and lastly 3′UTR (*n* = 28; 4.31%) (Figure 1E). On the contrary, the significant hypomethylated loci were not associated with any genes (*n* = 10,150; 39.58%) or in the gene body (*n* = 9211; 35.22%), while the rest were mainly located in TSS1500, 5′UTR, 3′UTR, TSS200, and 1st exon (Figure 1F). 

### 3.3. Methylation Level of Differentially Methylated Probes

**Figure 2:**
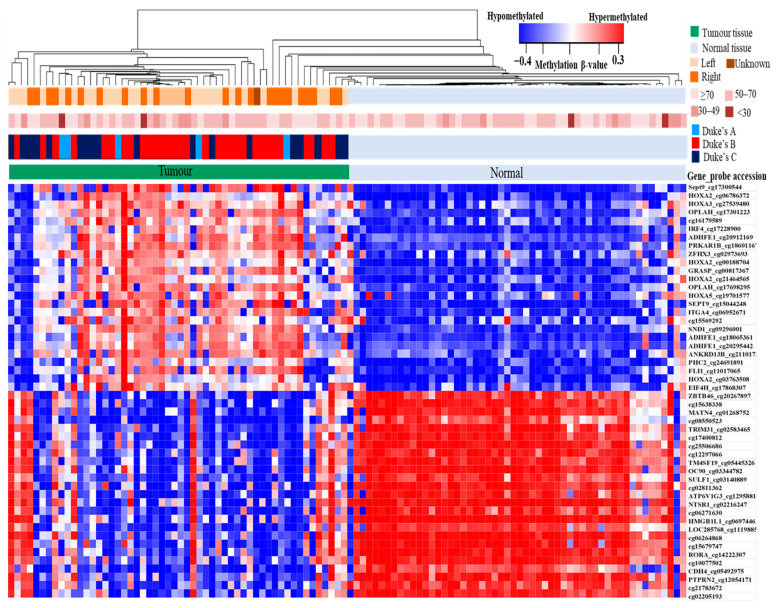
Heatmap of 50 differentially methylated genes, consisting of 25 hypermethylated and 25 hypomethylated genes in CRCs in comparison to the adjacent normal tissues with a *p*-value < 0.05. Every row represents individual genes, and the column represents individual samples. The horizontal bars indicate the patient’s age at diagnosis and tumour information, such as the tumour’s location and stage. The colour in each small box constitutes the methylation level of the genes in which red boxes indicate genes with a high methylation level while blue boxes display genes with a low methylation level.

Significant methylation differences of the 50 topmost significant differentially methylated probes were generated and illustrated through the heatmap in Figure 2. The five hypermethylated probes with the highest Δβ values were *SEPT9* cg17300544 (Δβ = 0.353), *HOXA2* cg06786372 (Δβ = 0.342), *HOXA3* cg27539480 (Δβ = 0.330), OPLAH cg17301223 (Δβ = 0.317), and cg16179589 (Δβ = 0.315). The list of the top 10 hypermethylated probes is displayed in Table 2, and information on the top 50 is provided in Supplementary Table S1. On the other hand, the five hypomethylated probes with the highest reduction of methylation were *ZBTB46* cg20267897 (Δβ = −0.497), cg15638338 (Δβ = −0.497), *MATN4* cg01268752 (Δβ = −0.496), cg08550523 (Δβ = −0.495), and *TRIM31* cg02583465 (Δβ = −0.495). The list of the top 10 hypomethylated probes is displayed in Table 3, and information on the top 50 hypomethylated probes is provided in Supplementary Table S2.

**Figure 3:**
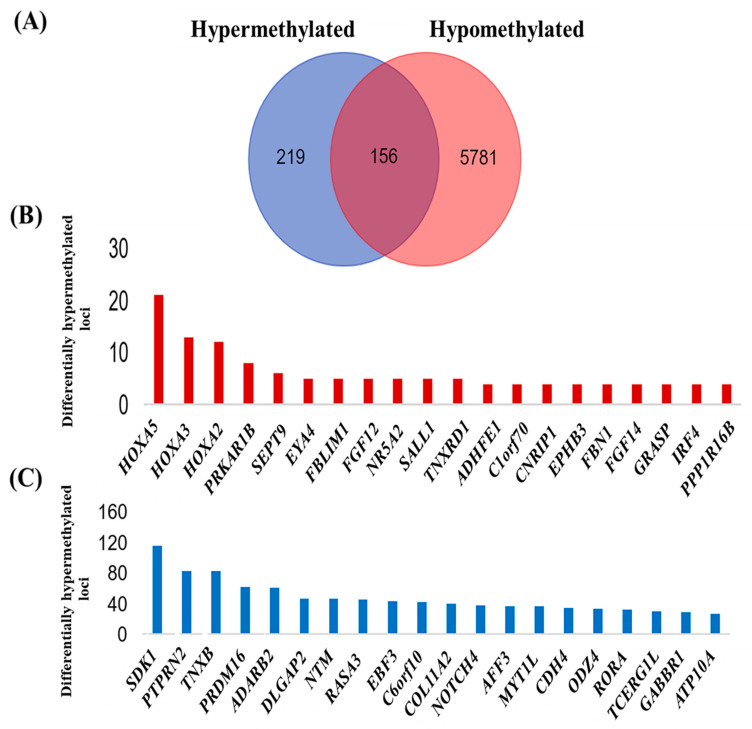
Overview of the methylation status identified in 6156 genes. (**A**) A total of 156 genes with overlapping methylation status and 5781 genes exhibit hypomethylation; in contrast, 219 genes were hypermethylated. (**B**) Bar chart displaying genes that had the highest differentially hypermethylated loci. (**C**) The bar chart details the genes that had the highest differentially hypomethylated loci.

Of the 26,093 differentially methylated CpGs identified from the probe-level test, 15,943 (61.1%) represented 6156 genes. There were 156 genes with overlapping methylation status; 5781 were uniquely hypomethylated, and 219 were uniquely hypermethylated (Figure 3A, Supplementary Table S3). The *HOXA5* gene had the highest number of differentially methylated loci (*n* = 21), followed by *HOXA3* (*n* = 13) and *HOXA2* (*n* = 12), in which all loci were hypermethylated (Figure 3B). Twenty loci were in the *HOXA5* islands, and one locus was at the shore. On the contrary, less than one-third of the hypermethylated loci in *HOXA3* were at the island; almost half (46%) were at the shore. *HOXA2* followed an almost similar trend, with the hypermethylated loci mainly at the shore and only one locus at the island. Another member of the *HOX* gene family, *HOXA6*, was also identified with three hypermethylated loci (data not shown). Other than these three genes, the majority (*n* = 284; 75.7%) of the genes had only one hypermethylated site.

There were more hypomethylated genes as compared to the number of hypermethylated genes. *SDK1*, *PTPRN2*, and *TNXB* were the genes with the highest number of hypomethylated loci (*n* = 116, 83, and 83, respectively) (Figure 3C). As expected, the majority of the hypomethylated loci in *SDK1* were at the open sea (81.8%), with *PTPRN2* and *TNXB* following a similar pattern.

### 3.4. Pathway Enrichment Analysis of DMG

Next, the list of hypermethylated and hypomethylated genes were subjected to pathway enrichment analysis using DAVID Functional Annotation Bioinformatics Microarray Analysis. We discovered ten enriched pathways of hypermethylated genes that could be potentially associated with CRCs. The top five most enriched pathways were pathways in cancer, the PI3K/Akt signalling pathway, signalling pathways regulating the pluripotency of stem cells, Proteoglycans in cancer, and Melanoma (Table 4). However, none of the listed pathways was statistically significant. Most of the genes that were hypermethylated in the pathways in cancer were *APC*, *CTBP2*, *NFKBIA*, *SMAD2*, *COL4A1*, *CDK6*, *DAPK2*, *FGF12*, *FGF14*, *FGF21*, *IGF1R*, *LAMB3,* as well as *PIK3R1*.

Conversely, the top five significantly enriched pathways associated with hypomethylated genes were the PI3K/AKT signalling pathway, pathways in cancer, focal adhesion, cell adhesion molecules, and the RAS signalling pathways (Table 5). Hypomethylated genes were observed in the PI3K/AKT signalling pathways, namely *CHRM2*, *TNXB*, *LAMA2*, *COL11A2*, *PIK3CD*, *PIK3CG*, *COMP*, *RPTOR*, *MYC*, *GNG7*, *PDGFD*, *AKT3*, *PDGFC*, *TNR, SYK*, *ANGPT1*, *ITGA4*, *IGF1*, *NGF*, *PTK2*, *RBL2*, *FGF14*, *CDK6*, *COL1A2*, *COL4A2*, *COL5A1*, *COL4A1*, *COL5A2*, *FGF18*, *COL6A3*, *COL6A6*, *SOS1*, *FGFR1*, and *CREB5.*

### 3.5. Integrated Analysis of Promoter Methylation and Gene Silencing

The hypermethylation of the promoter region has been associated with the silencing of the genes; meanwhile, cancer-linked DNA hypomethylation has received little attention due to the association with repeated DNA elements. We analysed the relationship between promoter hypermethylation and gene expression by integrating the differentially methylated genes (DMGs) and differentially expressed genes (DEGs). We identified a total of 105 hypermethylated genes in the promoter regions (comprised of transcription start sites; TSS1500 and TSS200 within the CG rich region).

To address whether the promoter methylation plays a role in the regulation of the gene expression, we observed the expression level of promoter hypermethylated genes using RNAseq data from The Cancer Genome Atlas (TCGA) of 134 CRCs and 23 normal colon samples. Out of 105 promoters hypermethylated, only 31 genes overlapped with DEGs from the TCGA datasets. The list of the promoter methylated genes, Δβ value, and corresponding expression level are displayed in Supplementary Table S4.

Then, from 31 overlapped genes, 28 genes exhibited hypermethylation associated with gene silencing in CRCs as compared to normal, whereas the three remaining genes displayed expression levels directly proportional to the methylation level.

**Figure 4:**
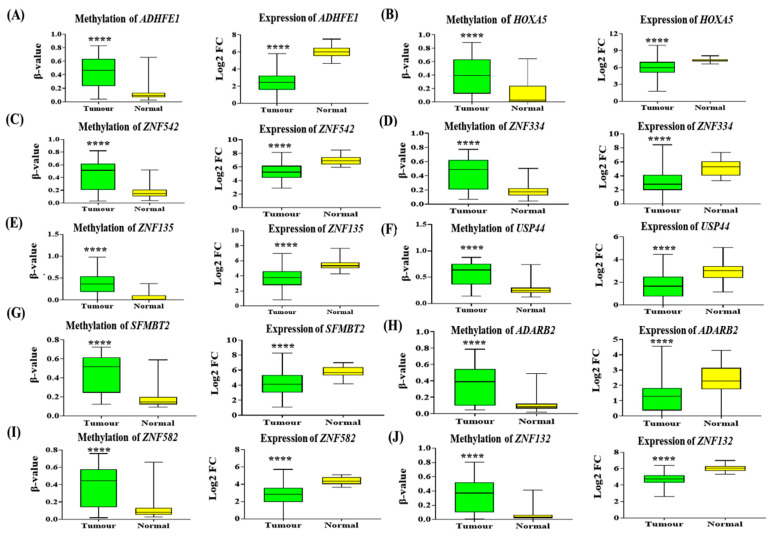
The methylation and expression level of the top ten hypermethylated genes are associated with gene silencing. The (**A**) *ADHFE1*, (**B**) *HOXA5*, (**C**) *ZNF542*, (D) *ZNF334*, (**E**) *ZNF 135*, (**F**) *USP44*, (**G**) *SFMBT2*, (**H**) *ADARB2*, (**I**) *ZNF582* and (**J**) *ZNF132* genes were significantly hypermethylated and downregulated in CRC tumours versus normal tissues. The box plot displays the average methylation level of each gene across 56 pairs of CRC tissues and their adjacent normal tissues from our microarray analysis. The green box plot represents the methylation level of the respective genes in CRC tumour tissues, while the yellow box plot represents normal colon tissues. The expression level of the genes in CRC tissues versus normal is shown on the right side of the methylation box plot graph, using 134 CRC tissues and 23 normal colon tissues from the TCGA dataset. The green box plot graph indicates the level of expression of the corresponding genes in CRC tissues, whereas the yellow box plot graph indicates the level of expression of the genes in normal tissues. The statistical analysis between tumour versus normal was determined using a two-sided Student’s t-test (**** *p*-value < 0.0001).

The top ten hypermethylated-induced silencing of genes in the promoter region were *ADHFE1* (Δβ = 0.299, log2 fold change = −3.531), *HOXA5* (Δβ = 0.271, log2 fold change = −1.419), *ZNF542* (Δβ = 0.261, log2 fold change = −1.675), *ZNF334* (Δβ = 0.259, log2 fold change = −1.887), *ZNF135* (Δβ = 0.258, log2 fold change = −1.720), *USP44* (Δβ = 0.255, log2 fold change = −1.225), *SFMBT2* (Δβ = 0.249, log2 fold change = −1.634), *ADARB2* (Δβ = 0.248, log2 fold change = −1.158), *ZNF582* (Δβ = 0.244, log2 fold change = −1.577), and *ZNF132* (Δβ = 0.244, log2 fold change = −1.379). This is illustrated in Figure 4.

### 3.6. Protein-Protein Interaction of Promoter Hypermethylated Genes

To explore the potential function of each protein encoded by the top ten promoter hypermethylated-silencing genes, we constructed a protein-protein interaction network using the STRING database Version 11.0 based on the homo sapiens association model. In this network, each node represents a protein, and each edge represents a physical interaction between two proteins.

The protein-protein interaction network of ADHFE1 resulted in 10 nodes and 18 edges with the enrichment *p*-value of 0.0181. The biological process of this protein is mainly involved in alcohol dehydrogenase activity, the retinol metabolism process, as well as ethanol oxidation.

Next, HOXA5 consisted of 10 nodes and 55 edges. The strong interaction of HOXA5 with other proteins provided the most significant enrichment *p*-value of 1 × 10^−^^16^. This protein is involved in DNA binding transcription activator activity, embryonic skeletal system morphogenesis, and development. The following protein, SFMBT2, was connected to 10 proteins and 13 edges. The protein-protein interaction enrichment *p*-value of SFMBT2 was 0.224, and it is involved in chromatin binding, histone binding, as well as transcription corepressor activity.

The zinc-finger (ZNF) proteins ZNF135, ZNF582, ZNF132, and ZNF334 consisted of 11 nodes and 15 edges, 11 nodes and 14 edges,11 nodes 12 edges with an enrichment *p*-value of 0.112, 0.15, 0.337, respectively. Among the zinc-finger families, ZNF334 displayed the least interaction with neighbouring proteins with 6 nodes and 5 edges, and the enrichment *p*-value of this protein was 0.572. ZNF families are involved in transcriptional regulation, ubiquitin-mediated protein degradation, signal transduction, and other cellular processes.

**Figure 5:**
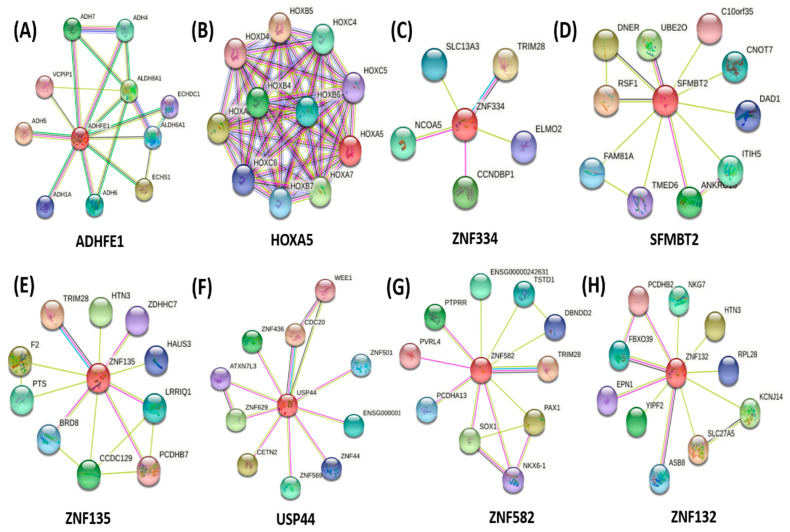
Interaction network of eight selected proteins from promoter hypermethylated-gene silencing. STRING Database Version 11.0b was used to construct the functional protein association networks based on the homo sapiens association model. Medium confidence was set to 0.400, and the max number of interactors was no more than 10. (**A**) The ADHFE1 protein is significantly connected to 10 neighbouring proteins with 18 edges, (**B**) The HOXA5 is strongly connected to 10 neighbouring proteins with 55 edges, (**C**) The ZNF 334 protein has the least interaction with neighbouring proteins with 6 nodes and 5 edges, (**D**) The SFMBT2 protein is connected to 10 neighbouring protein with 13 edges, (**E**) The ZNF135 protein shows interaction with 11 neighbouring proteins and 15 edges, (**F**) The USP44 protein is connected to 10 neighbouring proteins with 12 edges, (**G**) The ZNF582 interacts with 11 neighbouring proteins with 14 edges, (**H**) The ZNF132 protein interacts with 11 neighbouring proteins with 12 edges.

Another protein, namely USP44, is involved in regulating ubiquitin-protein ligase activity and cell division, specifically in the G2/M transition phase. USP44 protein was connected to 10 different proteins with 12 edges with an enrichment *p*-value of 0.353. On top of that, the protein interaction network of ADARB2 and ZNF542 could not be retrieved from the STRING database. Eight protein-protein interactions of promoter hypermethylated-silencing genes are displayed in Figure 5.

### 3.7. Receiver Operating Characteristics (ROC) Curve Analysis of Promoter Hypermethylated Genes

Next, to evaluate the diagnostic power of the genes as a biomarker, we performed the ROC analysis on the top ten promoter hypermethylated genes by measuring the specificity and sensitivity performance of the biomarkers (*p*-value < 0.0001) (Table 6). Among the top ten promoter hypermethylated genes, *ADHFE1* had the highest discriminative power (AUC = 0.9088, 95% CI = 0.847 to 0.971), followed by *SFMBT2* (AUC = 0.880, 95% CI = 0.818 to 0.942), and *ZNF135* (AUC = 0.859, 95% CI = 0.879 to 0.933).

In the previous analysis, we discovered *HOXA5* exhibited a strong protein-protein interaction network; however, the ROC analysis for *HOXA5* showed the lowest discriminative power with an AUC value of 0.776 to differentiate the CRC from normal mucosa tissues.

### 3.8. Methylation Level of ADHFE1 in Various Cancers

**Figure 6:**
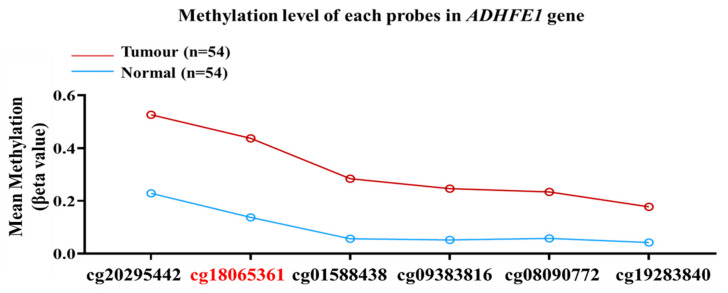
The methylation level of each probe in the promoter region of the *ADHFE1* gene in colorectal tumours and normal tissues. All probes were significantly hypermethylated in tumour versus normal, with cg18065361 (bold in red) having the highest methylation levels.

From the ROC analysis, we identified the potential of the *ADHFE1* gene as a diagnostic marker in CRC. All probes identified in the promoter region of the *ADHFE1* gene were hypermethylated, with cg18065361 exhibiting the highest methylation level in colorectal tumours versus normal tissues. The methylation status of the *ADHFE1* gene in each probe in the promoter region is illustrated in Figure 6.

Then, we performed an in silico methylation analysis of this gene in various cancers using Wanderer software [37] to compare the diagnostic potential of this gene in CRC as well as other cancers. This software provides the methylation level of all the Human Methylation 450K probes in the *ADHFE1* gene. Twenty-seven CpG loci in *ADHFE1* were found to be significantly aberrant methylated in 308 CRCs versus 38 normal tissues, including our CpG locus of interest; cg18065361.

**Figure 7:**
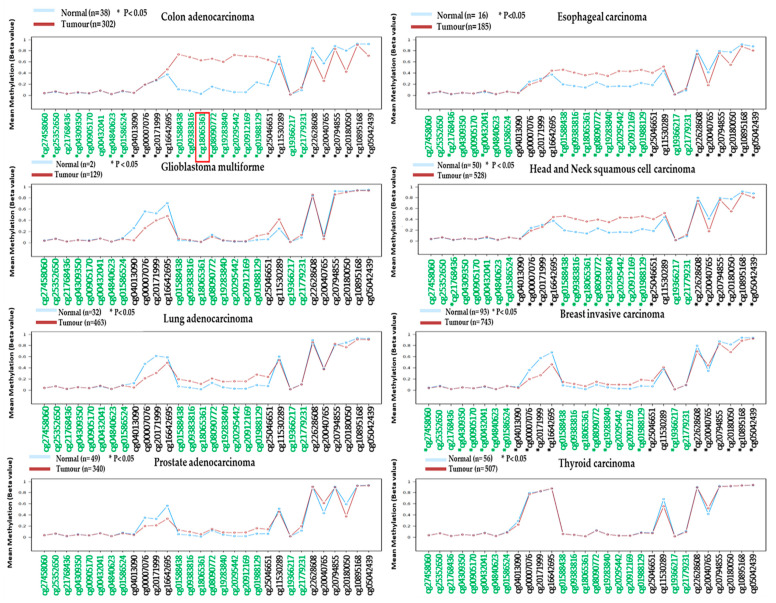
In silico validation of *ADHFE1* methylation in the TCGA dataset of selected cancers. The red box indicates the potential hypermethylated locus identified in our study. We displayed a concordant result with the TCGA dataset. Green probes indicate CpG island location, and the figure was generated using Wanderer software [36].

The methylation of *ADHFE1* cg18065361 was also significantly hypermethylated in esophageal carcinoma and head and neck squamous cell carcinoma. However, among these two cancers, CRC showed a significant difference in the methylation level of *ADHFE1* between tumours and normal tissues. Thus, based on our results and from the TCGA dataset, we concluded that the methylation of the *ADHFE1* gene occurs more frequently in CRC than in other malignancies. Figure 7 presents the methylation status of the *ADHFE1* gene in tumours versus normal tissues from various cancers using TCGA datasets.

## 4. Discussion

We analysed the global methylation status of 54 paired CRC and the corresponding normal tissue samples. Our demographic data showed that most patients were diagnosed over 50 years of age. Many studies have reported an increase in the incidence rate of CRC among individuals aged more than 50 years old [1,38]. To the best of our knowledge, this study provides the largest epigenome-wide DNA methylation profiles in CRC–adjacent normal colon tissue pairs using the 450K BeadChip. The Cancer Genome Atlas [39] has data on the DNA methylation status in 308 CRCs; however, only 38 matched tumour–adjacent normal samples were included. In 2013, Naumov and colleagues performed genome-wide methylation profiling in 22 paired CRC and adjacent normal tissues in addition to 19 colon tissue samples from cancer-free donors [40]. Recently, Gu et al. analysed 12 pairs of CRC and adjacent normal tissues using the newest version of methylation chip; the MethylationEpic Beadchip [41].

Our study revealed 26,093 differentially methylated probes that were distributed over the CpG sites of the genome. The CpG sites were comprised of several regions such as CpG island, shores (2 kb upstream from the island), shelves (2 kb upstream from the shores), and open sea, which make up for the remaining genomic region [42]. Notably, we discovered that most of the identified CpG sites were hypomethylated rather than hypermethylated, contrasting with previous findings in other cancers [43,44]. Our findings corroborated recent research by Gu and colleagues, which revealed that approximately 87% of differentially methylated CpG sites were hypomethylated, whereas just 13% were hypermethylated [41]. This may be explained by the genomic distribution of the HumanMethylation 450K microarray, which identified more probes in the open sea region (36.3%) than in CpG islands (30.9%), shores (23%), and shelves (9.7%) [45,46].

The CpG island is a region that is rich in CG sequences and often associated with the transcription start site. Our results showed that half of the hypermethylated probes were in the CpG island which is associated with promoter regions. This finding is supported by Sproul et al. where they showed that most of the cancers frequently exhibit hypermethylation at the CpG rich regions [47]. Conversely, hypomethylation often occurs in the open sea area of the genome. A similar pattern has been observed in another study where hypomethylation of CpG sites was enriched at the open sea area and intergenic region [48,49,50].

Various studies have reported septin 9 gene (*SEPT9*) methylation in CRC, highlighting the relevance of *SEPT9* methylation in cancer [51,52,53,54,55,56]. *SEPT9* is one of the widely studied hypermethylated genes in CRC, and our finding also supported its role in this cancer. We also uncovered many potential genes with interesting profiles. For instance, a group of Homeobox A (*HOXA*) cluster genes, the members of the HOX family, and an important gene in normal organ development was found to be significantly hypermethylated in CRC compared to the normal adjacent tissues. On top of that, four genes in this family, namely *HOXA5, HOXA3, HOXA2,* and *HOXA6,* were hypermethylated at multiple loci. Our discovery is supported by a recent study by Li and colleagues, who reported the hypermethylation of *HOXA5*, *HOXA2*, and *HOXA6* [57]. Furthermore, *HOXA5* methylation was shown to be associated with age, stage, and tumour status, while *HOXA6* methylation was linked to age and *KRAS* mutation [57]. The *HOXA* family has been the subject of substantial research in cancer. Numerous clinical trials have been conducted on the *HOXA* genes, but none have focused exclusively on the *HOXA2* gene. Recently, the methylation level of *HOXA1* was used to accurately differentiate between cholangiocarcinoma and benign biliary stricture from brushed biliary samples in clinical trial NCT04568512 [58]. *HOXA9* is among the biomarkers studied in the myeloid leukaemia clinical trial NCT03701295. The expression level of *HOXA9* was measured in myeloid leukaemia after treatment with chemotherapeutic drugs, including Pinometostat and Azacitidine [59]. Despite the fact that several *HOXA* family genes have been translated into clinical trials, none have focused entirely on the *HOXA2* gene. The relationship of *HOXA2* with cancer progression is limited, and the role of *HOXA2* in cancer prognosis and response to treatment is unknown. In 2019, Li and colleagues established a link between *HOXA2* and age, cancer staging, lymphovascular invasion, and lymph node involvement in CRC [57]. However, this is the only study within the last decade that revealed the significance of *HOXA2* in the clinicopathological characteristics of CRC. The identification of hypermethylation of the *HOXA2* gene as a biomarker for CRC in our study adds to the evidence of *HOXA2*′s association with cancer. Hence, our research may aid in the clinical development of *HOXA2*. In addition, it will be interesting to assess the clinicopathological correlation with the methylation status of *HOXA2* genes in our patients, which will be a subject for future research.

Human 5-oxoprolinase, *OPLAH,* was shown to be hypermethylated in our study, which had a significant impact on the gene’s downregulation, suggesting a possible contribution to CRC through the dysregulation of gene expression. Numerous investigations have also revealed that *OPLAH* is frequently hypermethylated in CRC versus normal tissues [40,60]. Despite a paucity of information on *OPLAH* methylation in cancer, several patents have been filed for its applicability in cancer detection. Recently, *OPLAH* was identified as one of the biomarkers that have been patented for the diagnosis of lung cancer (patent number: US 11028447 B2) [61] and CRC (patent number: US 11078539 B2) [62]. Taken together, our findings suggest that hypermethylated *OPLAH* has a role in the identification of CRC in the Malaysian population. In the future, the methylation status of *OPLAH* can be determined in blood, urine, and saliva, thereby establishing *OPLAH* as a non-invasive biomarker and accelerating the translation of molecular evidence to clinical practice.

Next, we discovered that *TRIM31* displayed a global loss of DNA methylation in CRC tissues. DNA hypomethylation is a common epigenetic alteration observed in human oncogenes. *TRIM31* is an oncogene that has been shown to be overexpressed in different types of cancer, including pancreatic [63], acute myeloid leukaemia [64], hepatocellular carcinoma [65], breast [66], and CRC [67]. Whilst *TRIM31* is commonly overexpressed in cancers, the mechanism by which *TRIM31* is overexpressed remains unknown. Our study is the first to demonstrate that *TRIM31* is hypomethylated in CRC. DNA hypomethylation may enhance gene expression by allowing transcription factors to bind to the promoter region of the gene. Therefore, we postulated that the overexpression of *TRIM31* in CRC is mediated by a global loss of DNA methylation. Nonetheless, additional research is necessary to confirm the association between DNA hypomethylation and *TRIM31* overexpression. In addition, increased *TRIM31* expression was associated with an aggressive phenotype and poor prognosis in pancreatic cancer. Moreover, this gene lowered the chemosensitivity of pancreatic cancer to gemcitabine, a commonly used chemotherapy treatment for pancreatic cancer [63]. In CRC, the high expression of *TRIM31* promotes cancer invasion and metastasis [67]. According to the findings presented, the overexpression of *TRIM31* had a role in the development, invasion, and metastasis of cancer, as well as resistance to chemotherapy.

Barrow and colleagues [68] performed an epigenome-wide analysis of DNA methylation in CRC patients with different smoking statuses, and among the significant hypomethylated genes were the *SDK1, PTPRN2,* and *TNXB* genes. In our study, we identified 116 hypomethylated *SDK1* loci and 83 hypomethylated loci in each of the *PTPRN2* and *TNXB* genes. These three genes also contained the highest number of hypomethylated loci. Sidekick cell adhesion molecule 1 (*SDK1*) hypomethylation was also reported in sporadic colorectal cancer [69] and is concordant with our findings. Receptor-type tyrosine-protein phosphatase N2 (*PTPRN2*) hypomethylation, on the other hand, has been rarely reported. The *TNXB* (tenascin XB) gene was first implicated in Ehlers–Danlos syndrome [70], but its role in several human cancers have been established, including nasopharyngeal [71] and mesothelioma [72]. Recent evidence further supports its role in malignancy, whereby *TNXB* is indicated as one of the triple-evidenced genes, which exhibit aberrant methylation, differentially expressed and associated with somatic mutation, hence, displaying the superior predictive ability in cancer diagnosis and prognosis [73].

The relationship between DNA methylation and gene expression is highly complex, and traditionally, DNA methylation-silenced gene expression was primarily affected in the CpG island promoter region [74,75,76] since the regulation of expression is controlled by transcription factors that bind to the promoter [77]. To gain further insight into the role of promoter methylation in silencing the gene expression, we performed an in silico analysis of gene expression profiles of normal and CRC samples from the TCGA datasets. We focused on the methylation profile of the promoter methylated genes and the association with the gene expression. Our analysis showed supporting evidence that genes associated with hypermethylated promoters display reduced gene expressions in CRC patients [78,79]. The addition of a methyl group at the promoter of the genes inhibits the binding of the transcription factor to the promoter region, hence, initiating the activation of genes [80].

A protein-protein interaction network was constructed to identify the regulator protein among the selected promoter hypermethylated. *HOXA5* and *ADHFE1* had strong protein-protein interactions with neighbouring proteins to form a regulatory network. The strong regulatory protein may influence the expression of neighbouring proteins, which contribute to the pathogenesis of CRC. Furthermore, the strong connection among the proteins is likely to form a protein complex and often direct the biological processes [81,82]. Our findings discovered the interaction of *HOXA5* with *HOXB6* and *HOXB7*. The downregulation of *HOXA5* increased the expression of *HOXB6* and *HOXB7,* which were associated with poor clinical outcomes in cancer patients [83,84,85]. On the other hand, the hypermethylation of *ADHFE1* further reduced the expression of neighbouring proteins, *ADH6*, *ADH7,* as well as *ADH1A,* in which the genes were associated with the patient’s prognosis and cancer pathogenesis [86,87,88].

Taken together, this summarises the potential of *HOXA5* and *ADHFE1* as promising biomarkers in CRC. However, when we performed ROC analysis for the top ten hypermethylated promoters mediating gene silencing, *ADHFE1* showed the highest discriminative values that significantly differentiated the CRCs from the normal colonic tissues. Compared with *HOXA5*, the better discrimination of normal and cancer tissues by *ADHFE1* signifies the great potential for this gene as a methylation marker to indicate pathological changes. *ADHFE1,* known as Alcohol Dehydrogenase Ion Containing 1, is a member of the iron-activated alcohol dehydrogenase family [89]. Consistent with previous studies, we observed that the CpG island methylation status of the *ADHFE1* promoter was higher in CRC tissues in contrast to their adjacent normal mucosa, and the loss expression of *ADHFE1* in CRC was associated with promoter methylation [90,91].

Our additional methylation analysis using the Wanderer database further strengthens the diagnostic potential of hypermethylated *ADHFE1* in detecting CRC as we observed that the methylation of *ADHFE1* displayed significant differences in CRCs versus normal tissues as compared to other cancers, for instance, glioblastoma multiforme (GBM), breast-invasive carcinoma, esophageal carcinoma, lung adenocarcinoma, head and neck squamous cell carcinoma, prostate adenocarcinoma, and thyroid carcinoma. The potential of *ADHFE1* as early detection of CRC was also discovered by Fan and his colleagues, whereby they observed hypermethylated *ADHFE1* in colorectal adenoma [92]. According to Moon et al., the hypermethylation of the *ADHFE1* gene promotes cell proliferation in CRC [91]. This finding was supported by Hu and colleagues, who demonstrated that the hypermethylation of the *ADHFE1* gene enhances CRC proliferation via altering cell cycle progression [89]. *ADHFE1* acted as a tumour suppressor gene in esophageal squamous cell carcinoma and was reported to be hypermethylated in a Chinese Han population [93]. More recently, the downregulation of the *ADHFE1* gene has been linked to decreased cancer survival [94]. Additionally, this study discovered that *ADHFE1* might contribute to cancer progression through its interactions with signalling pathways such as energy metabolism, DNA replication, and the cell cycle. With the evidence provided, we believed the promoter methylation-mediated downregulated gene of *ADHFE1* could be one of the potential DNA methylation biomarkers in detecting CRC. However, the diagnostic roles of *ADHFE1* will be subjected to further validation in a larger patient cohort in our country. It will be interesting if the methylation of *ADHFE1* can be detected in the blood for an early, accurate, and non-invasive blood-based biomarker.

## 5. Conclusions

This relatively extensive methylation study has revealed several potentially important genes in CRC that may be potential biomarker candidates. The hypomethylated and hypermethylated genes reported in this study are relevant to carcinogenesis and are in concordance with other studies. We also offer the first evidence for the potential of *HOXA2* cg06786372 *OPLAH* cg17301223, cg15638338, and *TRIM31* cg02583465 as diagnostic biomarkers for CRC. On top of that, we also discovered the potential of promoter methylation *ADHFE1* as a biomarker for CRC diagnosis. This is also the first insight into the epigenome-wide methylation profile of the cancer-adjacent normal colon in Malaysian CRC patients to complement the majority of data available from other populations. The new knowledge from this study can be utilised to advance our understanding of CRC methylomics; however, some of the findings need further investigations to confirm the involvement of the candidate genes in CRC.

## Figures and Tables

**Table 1:** Demographic data of the 54 CRC patients.

Characteristics	Number of Patients
**Gender**	
Female	31
Male	23
**Age (years)**	
≤50	8
>50	46
**Ethnicity**	
Malay	26
Chinese	24
Indian	4
**Duke’s Staging**	
A	5
B	31
C	18
**Tissue differentiation**	
Well	21
Moderately	27
Poorly	3
Unknown	3
**Location of the tumour**	
Right	24
Left	30

**Table 2:** Top 10 significant hypermethylated loci in CRC versus normal tissues.

Gene	Probes	Δβ	Genomic Feature	Genomic Region
*SEPT9*	cg17300544	0.353	TSS200	Island
*HOXA2*	cg06786372	0.342	Body	Shore
*HOXA3*	cg27539480	0.330	3′UTR	Shore
*OPLAH*	cg17301223	0.317	Body	Island
na	cg16179589	0.315	IGR	Shore
*IRF4*	cg17228900	0.314	5′UTR	Island
*ADHFE1*	cg20912169	0.311	5′UTR	Island
*PRKAR1B*	cg18601167	0.310	5′UTR	Shore
*ZFHX3*	cg02973693	0.308	5′UTR	Shelf
*HOXA2*	cg00188704	0.307	Body	Shelf

na = not available. The probes in the IGR region were not annotated with a gene name.

**Table 3:** Top 10 significant hypomethylated loci in CRC versus normal tissues.

Gene	Probes	Δβ	Genomic Feature	Genomic Region
*ZBTB46*	cg20267897	−0.497	5′UTR	Shore
na	cg15638338	−0.497	IGR	Open sea
*MATN4*	cg01268752	−0.496	Body	Shore
na	cg08550523	−0.495	IGR	Open sea
*TRIM31*	cg02583465	−0.495	Body	Open sea
na	cg17400812	−0.493	IGR	Open sea
na	cg25506686	−0.483	IGR	Open sea
na	cg12297066	−0.477	IGR	Open sea
*TM4SF19*	cg05445326	−0.476	TSS1500	Open sea
*OC90*	cg03344782	−0.470	Body	Open sea

na = not available. The probes in the IGR region were not annotated with a gene name.

**Table 4:** Five top pathways regulated by the hypermethylated genes in CRCs compared to adjacent normal tissues.

Pathway	No. of Genes	*p*-Value	Fold Enrichment
Pathways in cancer	14	0.6873	1.9923
PI3K/Akt signalling pathway	13	0.6873	2.1073
Signalling pathways regulating pluripotency of stem cells	10	0.1544	3.9947
Proteoglycans in cancer	9	0.6960	2.5167
Melanoma	6	0.6873	4.7262

**Table 5:** Five top pathways regulated by the hypomethylated genes in CRCs compared to adjacent normal tissues.

Pathway	No. of Genes	*p*-Value	Fold Enrichment
PI3K/Akt signalling pathway	34	2.89 × 10^−5^	2.6902
Pathways in cancer	28	0.0254	1.9448
Focal adhesion	23	3.73 × 10^−4^	3.0477
Cell adhesion molecules (CAMs)	21	2.89 × 10^−5^	4.0369
Ras signalling pathway	19	0.0314	2.2949

**Table 6:** Receiver operating characteristics (ROC) curve analysis between CRCs and normal samples of promoter hypermethylated genes.

Gene	Probes	AUC	95% Confidence Interval	*p*-Value
*ADHFE1*	cg18065361	0.909	0.8470–0.9706	<0.0001
*HOXA5*	cg19643053	0.776	0.6876–0.8639	<0.0001
*ZNF542*	cg27477373	0.844	0.7656–0.9230	<0.0001
*ZNF334*	cg10140114	0.826	0.7432–0.9090	<0.0001
*ZNF135*	cg06454760	0.859	0.7855–0.9326	<0.0001
*USP44*	cg13879483	0.829	0.7445–0.9140	<0.0001
*SFMBT2*	cg02866454	0.880	0.8180–0.9420	<0.0001
*ZNF582*	cg13916740	0.809	0.7198–0.8982	<0.0001
*ZNF132*	cg03735888	0.842	0.7654–0.9184	<0.0001
*ADARB2*	cg02899206	0.79	0.7055–0.8810	<0.0001

## Data Availability

The raw data of microarray methylation can be obtained in GEO under accession GSE193535.

## Supplementary Materials

The following are available online at https://www.mdpi.com/article/10.3390/diagnostics12010198/s1. Table S1: Top 50 hypermethylated genes in CRC compared to the normal adjacent colon, Table S2: Top 50 hypomethylated genes in CRC compared to the normal adjacent colon, Table S3: List of differentially methylated genes and Table S4: The list of promoter hypermethylation genes with Δβ value and their corresponding expression level.
